# PacBio Single-Molecule Long-Read Sequencing Provides New Light on the Complexity of Full-Length Transcripts in Cattle

**DOI:** 10.3389/fgene.2021.664974

**Published:** 2021-08-30

**Authors:** Tianpeng Chang, Bingxing An, Mang Liang, Xinghai Duan, Lili Du, Wentao Cai, Bo Zhu, Xue Gao, Yan Chen, Lingyang Xu, Lupei Zhang, Huijiang Gao, Junya Li

**Affiliations:** ^1^Laboratory of Molecular Biology and Bovine Breeding, Institute of Animal Science, Chinese Academy of Agricultural Sciences, Beijing, China; ^2^College of Animal Science and Technology, Southwest University, Chongqing, China

**Keywords:** cattle, PacBio single-molecule long-read sequencing, full-length transcript, alternative splicing, alternative polyadenylation, long non-coding RNA

## Abstract

Cattle (*Bos taurus*) is one of the most widely distributed livestock species in the world, and provides us with high-quality milk and meat which have a huge impact on the quality of human life. Therefore, accurate and complete transcriptome and genome annotation are of great value to the research of cattle breeding. In this study, we used error-corrected PacBio single-molecule real-time (SMRT) data to perform whole-transcriptome profiling in cattle. Then, 22.5 Gb of subreads was generated, including 381,423 circular consensus sequences (CCSs), among which 276,295 full-length non-chimeric (FLNC) sequences were identified. After correction by Illumina short reads, we obtained 22,353 error-corrected isoforms. A total of 305 alternative splicing (AS) events and 3,795 alternative polyadenylation (APA) sites were detected by transcriptome structural analysis. Furthermore, we identified 457 novel genes, 120 putative transcription factors (TFs), and 569 novel long non-coding RNAs (lncRNAs). Taken together, this research improves our understanding and provides new insights into the complexity of full-length transcripts in cattle.

## Introduction

Cattle (*Bos taurus*) are an agriculturally important species that provide human beings with large quantities of high-quality protein. As a typical ruminant animal, cattle still play a great role in sustainable agriculture since they can effectively utilize pastures, silage, and high-fiber crop residues. Nowadays, genomic information plays an important role in accelerating the molecular breeding process of cattle, so an accurate and complete reference genome and annotation are essential for genetic mechanism research, Quantitative trait locus (QTL) mapping, and genomic selection of important production traits for cattle breeding. The latest reference genome assembly (ARS-UCD1.2) was first reported in 2018, assembling 2.7 Gb of the genome (Rosen et al., 2020). The annotation of the ARS-UCD1.2 assembly (NCBI release 106), resulted in 21,039 protein-coding genes, 9,357 non-coding genes, and 4,569 pseudogenes (Rosen et al., 2020). This assembly has a higher sequence continuity and accuracy than the previous reference (UMD3.1.1), and the protein models predicted in ARS-UCD1.2 assembly annotation are generally more complete than in UMD3.1.1 annotation (Zimin et al., 2009; Rosen et al., 2020). However, due to the diversity of cattle breeds, there are distinct genetic characteristics and allelic variations between breeds, so more genomic information is needed to explore to complete the annotation of the structural and functional of the current cattle reference genome (Crysnanto and Pausch, 2020).

The next-generation sequencing (NGS) technologies, such as the Illumina platform, has stimulated the construction of genome and transcriptome resources for many species (Lan et al., 2012; Li et al., 2012; Oono et al., 2013; Du et al., 2017; Carruthers et al., 2018). NGS is accurate, cost-effective, and supported by a wide range of analysis software and pipelines. However, natural nucleic acid polymers span eight orders of magnitude in length, and sequencing them in short amplified fragments complicates the task of reconstructing and counting the original molecules (Conesa et al., 2016; Amarasinghe et al., 2020). Therefore, it is difficult to accurately reconstruct expressed full-length transcripts, predicting splice isoforms and analyzing the transcriptome diversity based on NGS reads (Wang L. et al., 2019). The third-generation sequencing (TGS) technologies, which include Pacific Biosciences single-molecule real-time (SMRT) and Oxford Nanopore Technologies (ONT) nanopore sequencing technology, can avoid the disadvantages of NGS technology and obtain high-quality long-read transcripts due to their ability to sequence reads up to 50 kb (Jia et al., 2018; Zuo et al., 2018). Then, we can obtain more complete transcripts and analyze structural variations in the genome and transcriptome (Eid et al., 2009; Koren et al., 2012; Sharon et al., 2013; Tilgner et al., 2015; Amarasinghe et al., 2020). However, the long-read sequencing technologies still have some limitations, such as higher error rates and relatively low throughput (Wang et al., 2016; Wang X. et al., 2019). Several studies have indicated that the error rate for SMRT sequencing (15%) is higher than the Illumina platform (1%) (Weirather et al., 2017; Amarasinghe et al., 2020). Moreover, NGS and TGS technologies have different error models. The Illumina short reads mainly contain miscalled bases with increasing frequency toward read ends, while SMRT sequencing generates primarily insertion-deletion errors in a random pattern (Hackl et al., 2014; Beiki et al., 2019). Fortunately, some research has shown that the accurate and abundant NGS reads can be used to correct errors in TGS and improve the accuracy of long reads sequencing (Sharon et al., 2013; Xu et al., 2015).

Recently, the strategy of combining SMRT sequencing and Illumina RNA-seq data to detect structural variation, novel genes, or isoforms and reveal functional variety at the transcriptional level has become more prevalent (Amarasinghe et al., 2020). According to this strategy, 24,797 alternative splicing (AS) and 11,184 alternative polyadenylation (APA) events were detected in rabbit (Chen et al., 2017). In pig, researchers detected 28,127 novel isoforms from 26,881 novel genes based on the high-quality full-length isoforms. Meanwhile, they identified more than 92,000 novel AS events and found intron retention (RI) and exon skipping (ES) were the main AS events in AS model (Li et al., 2018). Another pig study observed many unique transcripts and extended more than 6,000 known gene borders, and the extensions were verified by independent ChIP-seq, 3′-RNA-seq experiment, and human CAGE data (Beiki et al., 2019). In maize, many unique isoforms and higher isoform densities were detected with SMRT sequencing, and 867 novel lncRNAs were identified which had a much longer mean length than those identified by Illumina short-read sequencing (Wang et al., 2016). These findings have provided important information for improving genome annotation and gene models for different species.

To improve the transcriptional information and explore the complexity of cattle transcriptome, we generated high-quality FLNC reads in the present study by PacBio SMRT sequencing. We first used Illumina short reads to correct the relatively high error rates of SMRT long reads. Then, AS and APA events were detected to explore the structural complexity of transcripts. We predicted the novel genes and annotated them using seven databases, and the transcript factor (TF) and lncRNAs were investigated. Accordingly, our research contributed to the exploration of the splice isoforms and transcriptome diversity of cattle, increased our understanding of the structure of the transcript, and facilitates the further study of the genetics of cattle.

## Materials and Methods

### Collection of Samples and RNA Preparation

The sample collection experiment was conducted on the farm of Hanjiang Beef Cattle Co., Ltd. (Hubei, China). The use of animals and private land in this study was approved by their respective legal owners. The cattle were raised in the same feeding strategies and conditions. Three unrelated male Simmental beef calves were collected at 0 days of age. The calves were stunned by electrical shock and killed while unconscious. Then, six tissues, consisting of cerebrum, rumen, liver, spleen, renal cortical, and longissimus muscle, were sampled for each cattle, snap frozen, and stored in liquid nitrogen until use. Subsequently, all 18 samples were subjected to RNA extraction using TRIzol reagent (Takara, Dalian, China) according to the manufacturer’s instructions. The RNA concentration was measured using Qubit^®^ RNA Assay Kit in Qubit^®^ 2.0 Fluorometer (Life Technologies, Carlsbad, CA, United States). RNA integrity and purity were assessed using the Nanodrop ND-1000 spectrophotometer (NanoDrop Technologies, Wilmington, DE, United States) and RNA Nano 6000 Assay Kit of the Agilent Bioanalyzer 2100 system (Agilent Technologies, Palo Alto, CA, United States), respectively. Qualified RNA samples were then used for further cDNA library construction and sequencing.

### SMRT Library Preparation and PacBio Sequencing

One microgram for each RNA sample was equally pooled together and prepared for PacBio SMRT library construction. Full-length cDNA was synthesized by use of the Clontech SMARTer PCR cDNA Synthesis Kit (TaKaRa, Dalian, China), and cDNA fraction and length selection (<4 kb and >4 kb) was performed using the BluePippin^TM^ Size Selection System (Sage Science, Beverly, MA, United States). Then, one SMRT bell library was generated using the Pacific Biosciences DNA Template Prep Kit 2.0 (Pacific Biosciences, CA, United States) according to the standard method. All the samples were pooled onto one SMRT cell library, so SMRT data of different tissues were not available, and tissue-specific analysis of isoforms was not possible. Finally, SMRT sequencing was performed on the Pacific Bioscience Sequel platform.

### Illumina cDNA Library Construction and NGS Analysis

All the 18 RNA samples were prepared for Illumina unstranded cDNA library construction. Briefly, polyadenylated RNA was isolated and fragmented into ∼200 bp fragments. The first and second cDNA strands were synthesized successively. The repaired and purified double-stranded cDNA fragments were selected by size. Then, the qualified and amplified mRNA libraries were finally sequenced on an Illumina NovaSeq 6000 platform, and 150 bp paired-end raw short reads were generated. The raw short reads were subjected to quality filtering using NGS QC Toolkit (v2.3.3) (Patel and Jain, 2012), for which we trimmed the first five bases from the 5′ end of the reads and removed reads consisting of the low-quality bases (QA ≤ 30) >20% or ambiguous bases >1%. To produce corrected PacBio long reads, Illumina clean reads are used for independently assembling transcripts using Hisat2 (v2.1.0) and Stringtie (v2.1.1) (Kim et al., 2015; Pertea et al., 2015).

### Quality Filtering and Error Correction for PacBio Long Reads

The PacBio raw data were processed using the SMRTlink (v7.0) software with parameters: minReadScore = 0.75, minLength = 200. Circular consensus sequences (CCSs) were generated from subread BAM files (parameters: min_length 200, max_drop_fraction 0.8, no_polish TRUE, min_zscore –9999, min_passes 2, min_predicted_accuracy 0.8, max_length 18,000) and then a BAM file of CCS was generated. By searching for the 5′ and 3′ adapters and the poly(A) tail, the CCS was classified into full length and non-full length (NFL) reads. Full-length reads with all the three elements and any additional copies of the adapter sequence within the DNA fragment were classified as FLNC. We then used ICE (Iterative Clustering for Error Correction) to identify the consensus isoforms that formed FLNC and polished the consensus isoforms with NFL reads to obtain high-quality isoforms with post-correction accuracy above 99% using Quiver (parameters: hq_quiver_min_accuracy 0.99, bin_by_primer false, bin_size_kb 1, qv_trim_5p 100, qv_trim_3p 30). Next, the Illumina clean data generated above was used to correct nucleotide indels and mismatches in consensus reads with the LoRDEC software (v0.7) (Salmela and Rivals, 2014). LoRDEC uses a hybrid error correction strategy that builds a succinct de Bruijn graph representing the Illumina short reads, and seeks a corrective sequence for each erroneous region in the PacBio long reads by traversing chosen paths in the graph (Fu et al., 2019). Then, a high-quality PacBio corrected consensus reads dataset without redundant isoforms was constructed. Finally, we used the method proposed by Salmela and Rivals (2014) to evaluate the PacBio data error rate before and after error correction.

### Mapping to the Reference Genome and Structural Analysis of Genes

Corrected isoforms were aligned to the cattle reference genome (ARS-UCD1.2) with the Genome Mapping and Alignment Program (GMAP, version: 2017-06-20) using the following parameters: –no-chimeras, –cross-species, –expand-offsets 1 -B 5 -K 50000 -f samse -n 1 (Wu and Watanabe, 2005). The genome annotation file (NCBI release 106) was used for gene and transcript determination. Genome-guided construction of the full transcriptome was successful. Transcripts structure analysis was performed using the TAPIS pipeline (Version 1.2.1) (Abdel-Ghany et al., 2016). AS events including IR, ES, alternative 3′ splice site (Alt.3′), alternative 5′ splice site (Alt.5′), mutually exclusive exon (MEE), alternative first exons (AF), and alternative last exons (AL) were identified and classified using SUPPA (v2.3) (Alamancos et al., 2015). Among them, IR is defined as when one intron is retained within a longer exon and flanked by two shorter exons simultaneously. ES is defined as when an exon is absent in some transcripts but present in others. If an intron is excised at more than one site and linked to its 5′ or 3′ exons with different boundaries, they are considered as the Alt. 5′ and Alt. 3′ (Chen et al., 2017). Alternative terminal exon regulations including AF or AL are types of AS, which couples with alternative transcription start sites and APA, respectively (Lian et al., 2020). APA events were analyzed by TAPIS described previously. The transcription factors (TFs) were predicted using the animalTFDB 2.0 database (Zhang et al., 2015).

Due to the limitation of library construction, we can only obtain lncRNA containing polyA tails. The following four tools were combined: Coding Potential Calculator (CPC) (Kong et al., 2007), Coding–Non-Coding Index (CNCI) (Sun et al., 2013), PLEK (Li et al., 2014), and Pfam database (Finn et al., 2016). They were used to sort non-protein-coding RNA candidates from putative protein-coding RNAs in the transcripts. Putative protein-coding RNAs were filtered out using minimum length and exon number thresholds. The transcripts longer than 200 bp with more than two exons were selected as lncRNAs candidates and then screened using CPC/CNCI/PLEK/Pfam, as these tools can distinguish protein-coding from the non-protein-coding genes. Only the transcripts identified in the four databases were regarded as lncRNAs.

### Novel Gene Prediction and Functional Annotation

Here we defined a novel (compared to NCBI gene-build) gene as a gene putatively encoding a detected transcript that does not match any annotated gene in the cattle reference genome (ARS-UCD1.2). To obtain comprehensive annotation information, functional annotations of the novel genes were conducted using the following seven databases: NR (NCBI non-redundant protein sequences); NT (NCBI non-redundant nucleotide sequences); Pfam (Protein family); KOG (EuKaryotic Ortholog Groups of proteins) (Tatusov et al., 2000); Swiss-Prot (a manually annotated and reviewed protein sequence database); KO (KEGG Ortholog database) (Kanehisa et al., 2004); and GO (Gene Ontology). We used the software of BLAST and set the *e*-value “1e-10” in the NT database analysis. We used the software of Diamond BLASTX and set the *e*-value “1e-10” in the NR, KOG, Swiss-Prot, and KEGG database analyses. We used the software of Hmmscan in the Pfam database analysis. For each transcript searched in the four databases, functional information for the best-matched sequence was assigned to the query transcript.

## Results

### General Properties of PacBio Sequencing

**FIGURE 1:**
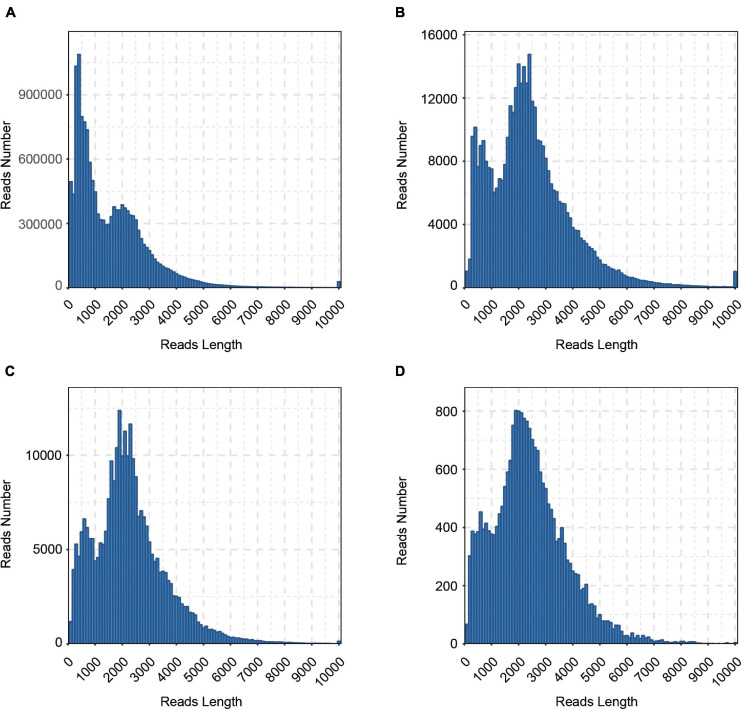
Length distributions of PacBio SMRT sequencing. **(A)** Number and length distributions of 14,750,730 Subreads sequences. **(B)** Number and length distributions of 381,423 CCS sequences. **(C)** Number and length distributions of 276,295 FLNC sequences. **(D)** Number and length distributions of 22,353 corrected sequences.

To reveal the complexity of the transcriptome in cattle, six tissues (cerebrum, rumen, liver, spleen, renal cortical, and longissimus muscle) were collected and a pooled RNA sample of them was sequenced with the Pacific Bioscience Sequel platform to accurately capture full-length sequences and uncover full-length splice variants. With SMRT sequencing, 23.6 Gb of raw data consisting of 441,444 raw polymerase reads was generated. Then, a total of 14,750,730 subreads (22.5 Gb) were obtained, with an average read length of 1,526 bp and N50 of 2,367 bp. To provide more accurate sequence information, CCS was generated from subreads that pass at least 2 times through the insert, and a total of 381,423 CCSs were obtained. In all, 286,688 CCSs were identified as full-length reads, and 276,295 were identified as FLNC reads with low artificial concatemers. The mean length of FLNC reads was 2,241 bp. The length distribution of the subreads, CCSs, FLNC reads is shown in Figure 1 and Table 1.

**TABLE 1:** Summary of reads from PacBio SMRT sequencing.

	**Polymerase read**	**Subreads**	**CCS**	**FLNC**	**Corrected consensus**
Number	441,444	14,750,730	381,423	276,295	22,353
Mean length	53,457	1,526	2,396	2,241	2,379
N50	109,539	2,367	2,963	2,725	2,921

### Error Correction of PacBio Long Reads Using Illumina Reads

The FLNC reads with similar sequences were clustered together using the ICE (Iterative isoform-clustering) algorithm, and each cluster was considered as a consistent sequence. Combined with NFL sequences, the Quiver program was used to polish the consistent sequences in each cluster. To further correct the relatively high error rates of PacBio long reads, we generated ∼981.4 million clean reads of NGS sequencing clean data. Then, the Illumina short reads were used for correcting the consensus isoform sequences of PacBio long reads. The LoRDEC software was used to correct polished consensus sequences, resulting in 22,353 corrected sequences, with an N50 length of 2,921 bp and a mean read length of 2,379 bp (Table 1 and Figure 1D). To calculate the error rate, the raw and corrected PacBio long reads were aligned to the cattle reference genome (ARS-UCD1.2) with BLASR (Chaisson and Tesler, 2012). The error rate is defined as the sum of the numbers of bases of insertions, deletions, and substitutions in the alignment divided by the length of aligned regions for each read. After calculation, the error rates of PacBio long reads before and after error correction were 9.72 and 2.84%, respectively.

### Genome Mapping

**FIGURE 2:**
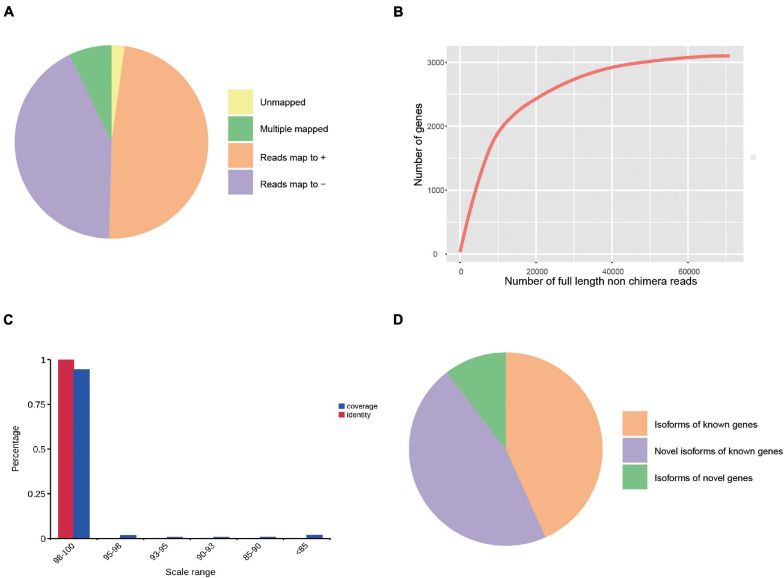
Genome Mapping and Alignment Program (GMAP) analysis of SMRT sequencing. **(A)** GMAP mapping statistics of the corrected sequences. **(B)** Saturation curve of corrected sequences, *x*-axis represents numbers of full-length non-chimera (FLNC) reads, *y*-axis represents numbers of genes. **(C)** Range of mapping coverage and identity, *x*-axis represents the scale ranges, *y*-axis represents the percentages. **(D)** Classification of transcript isoforms identified.

We compared all the corrected sequences against the cattle reference genome using GMAP software. A total of 21,863 reads (97.81%) were mapped to the reference genome. According to mapping results, these reads could be divided into four groups: unmapped, multiple mapped, mapped to “+” and mapped to “−” (Table 2 and Figure 2A). The unmapped group consisted of 490 reads (2.19%) with no significant mapping to the reference genome. The multiple mapped group consisted of 1,583 reads (7.08%) showing multiple alignments. The group of results mapped to “+” consisted of 10,778 reads (48.22%) that were mapped to the positive strand of the reference genome, and the group of results mapped to “−” consisted of 9,502 reads (42.51%) which were mapped to the opposite strand of the reference genome. The curve of the corrected isoform numbers reached a saturation level (Figure 2B), and high-quality reads (i.e., with coverage and identity values over 98%) accounted for over 90% (Figure 2C).

**TABLE 2:** Comparisons of PacBio and Illumina sequenced data for read mapping.

**Terms**	**PacBio sequenced data**		**Illumina sequenced data**	
	**Number of reads**	**Percentage (%)**	**Number of reads**	**Percentage (%)**
Total mapped	21,863	97.81	954,571,462	97.27
Multiple mapped	1,583	7.08	23,405,640	2.38
Uniquely mapped	20,280	90.73	931,165,822	94.88
Reads map to “+” strands	10,778	48.22	465,119,568	47.39
Reads map to “−” strands	9,502	42.51	466,046,254	47.49

### Gene Structure Analysis

**FIGURE 3:**
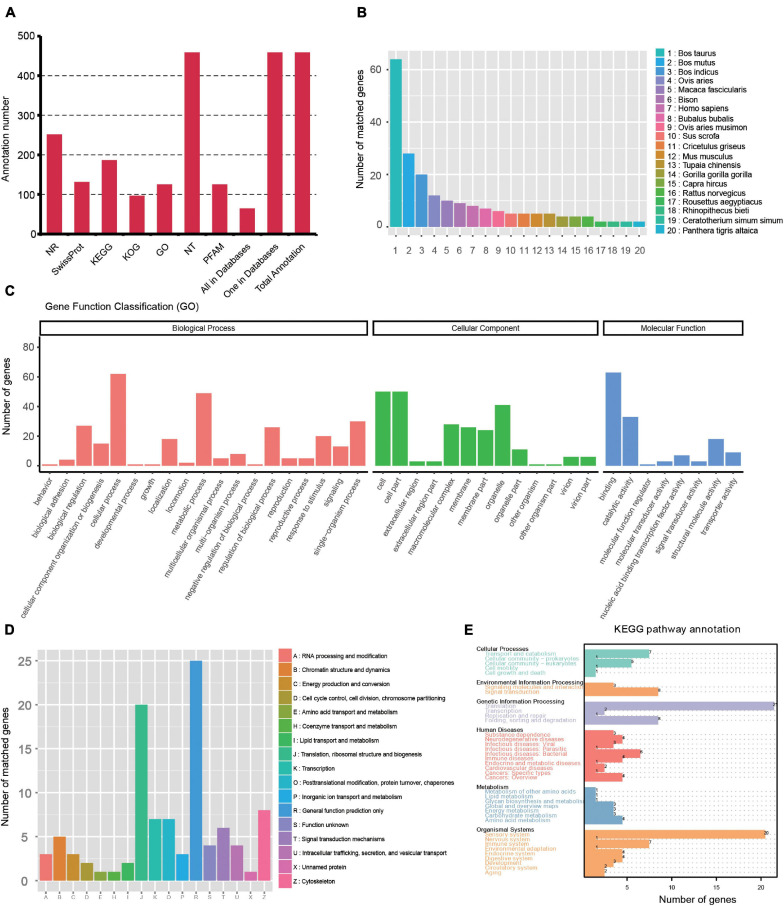
Function annotation of novel genes. **(A)** Function annotation of novel genes in all databases (NR, NT, Pfam, KOG, Swiss-Prot, KEGG, and GO). **(B)** Nr Homologous species distribution diagram of novel genes. **(C)** Distribution of GO terms for all annotated transcripts in biological process, cellular component, and molecular function. **(D)** KOG enrichment of novel genes. **(E)** KEGG pathways enrichment of novel genes.

Gene structure analysis was performed using the TAPIS pipeline. The GMAP output file and genome annotation (NCBI release 106) file were used for gene and transcript isoforms determination. Reads that were mapped to different exons in known gene regions were considered new isoforms, and isoforms spanning two or more genes are removed from downstream splice isoform analysis. Then, 4,862 isoforms were identified, and they can be divided into three types: (1) 2,104 isoforms of known genes; (2) 2,250 novel isoforms from known genes; and (3) 508 novel isoforms from novel genes (Figure 2D and Supplementary Table 1). Also, 457 novel genes (Supplementary Table 2) were identified and they were annotated using the Nt, Nr, Swissprot, GO, KOG, Pfam, and KEGG databases. A total of 63 genes had hits on all 7 databases, and 457 had hits on at least 1 database (Figure 3A and Supplementary Table 3). We analyzed homologous species by comparing the novel genes to the NR database, and the results showed that the largest five number of the novel genes were distributed in *Bos taurus* (64), *Bos mutus* (28), *Bos indicus* (20), *Ovis aries* (12), and *Macaca fascicularis* (10) (Figure 3B). By using all annotated genes in the cattle genome as background, GO analysis showed that “Cell,” “binding,” and “cellular process” were ranked as the most enriched items in the “cellular components,” “molecular functions,” and “biological process” categories, respectively (Figure 3C). KOG analysis showed the novel genes were assigned to 17 functional clusters, and the “general function prediction only,” “translation, ribosomal structure and biogenesis,” and “cytoskeleton” ranked as the top three largest categories (Figure 3D). The KEGG results demonstrated that the novel genes were mapped to 90 KEGG pathways (Figure 3E).

### Analysis of AS and APA Events

**FIGURE 4:**
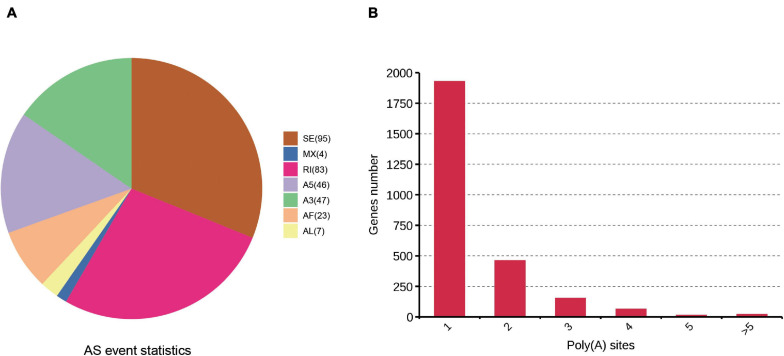
Identification of AS and APA events based on the SMRT sequencing. **(A)** Number and categories of the AS events identified. **(B)** Distribution of the number of poly(A) sites per gene.

One of the most important advantages of PacBio sequencing is its ability to identify AS events by directly comparing different isoforms of the same gene. Here, AS events were analyzed with SUPPA software. Seven AS events (IR, SE, Alt.3′, Alt.5′, MX, AF, and AL) were identified. A total of 305 AS events were found based on the PacBio SMRT reads (Supplementary Table 4). Two kinds of events, skipped exons (95) and retained introns (83), were much more common than other AS events (Figure 4A). PacBio sequencing also enables the investigation of the APA sites. In our study, 3,795 poly(A) sites were identified among the 2,643 genes in the cattle reference genome, 1,929 genes showed 1 poly(A) site, and 21 genes contained at least 5 poly(A) sites (Figure 4B and Supplementary Table 5). The average number of poly(A) sites per gene was 1.43.

### Identification of TF and lncRNA

**FIGURE 5:**
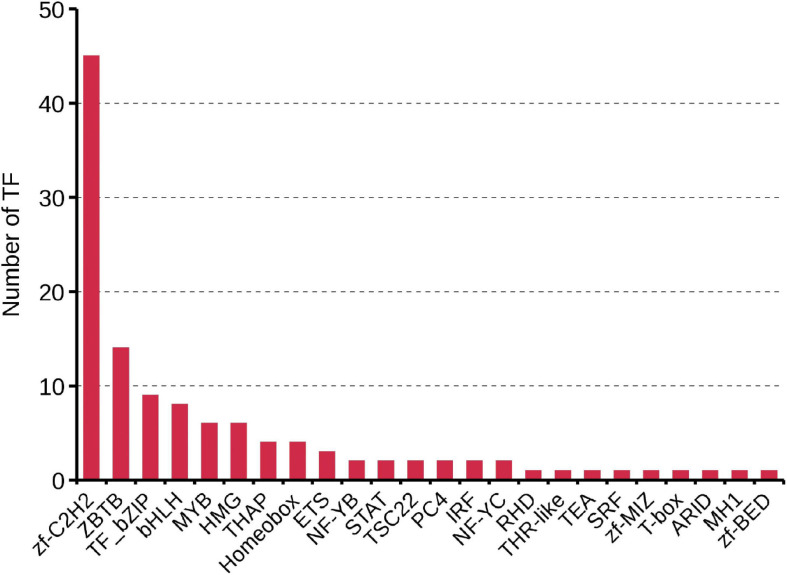
Identification of transcription factors based on the SMRT sequencing.

**FIGURE 6:**
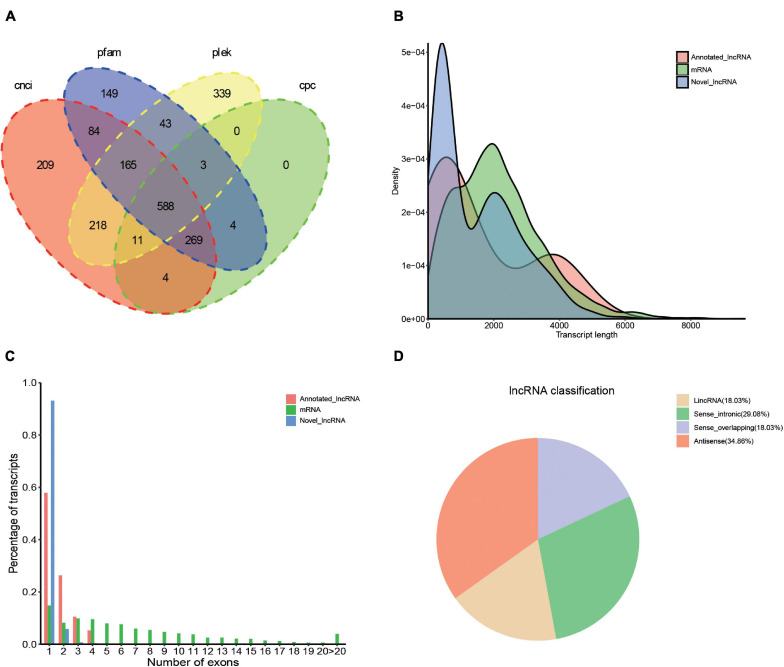
Identification of lncRNA based on the SMRT sequencing. **(A)** Venn diagram of lncRNA predicted by CNCI, CPC, PLEK, and Pfam tools. **(B)** Length and density distribution of annotated lncRNA, novel lncRNA, and mRNA identified. **(C)** Comparison of exon number of annotated lncRNA, mRNA, and novel lncRNA. **(D)** Classification of the types of lncRNA.

Transcription factors (TFs) play important regulatory roles in animal growth and development. In this study, they were identified and classified with the animalTFDB 2.0 database. A total of 120 putative TFs from 24 families were identified, of which 11 TFs were identified as novel. The numbers of TFs enriched were as follows: zf-C2H2 (45), ZBTB (14), TF_bZIP (9), bHLH (8), and MYB (6) (Figure 5 and Supplementary Table 6). Based on the prediction of CPC, CNCI, PLEK, and Pfam databases, 2086 transcripts were considered as putative non-coding RNAs. Finally, 588 transcripts found in all 4 prediction results were considered as lncRNAs and 569 (96.8%) of them were novel lncRNAs (Figure 6A and Supplementary Table 7). Length distribution analysis of the lncRNAs revealed that their lengths ranged from 0.2 to 7.65 kb and the mean length was 1.51 kb (Figure 6B). The lncRNAs predicted have fewer exons when compared to the mRNAs and 541 (92%) of the lncRNAs were single exons (Figure 6C). Additionally, the identified lncRNAs were further classified into four types, including 205 antisense lncRNAs (34.86%), 171 sense intronic lncRNAs (29.08%), 106 sense overlapping lncRNAs (18.03%), and 106 lincRNAs (18.03%) (Figure 6D).

## Discussion

RNA sequencing (RNA-seq) has become a ubiquitous tool for transcriptome-wide analysis of differential gene expression and transcript structure. However, the major limitation of short-read is the difficulty in accurately reconstructing expressed full-length transcripts from the assembly of reads, which are useful for functional studies of important genes (Conesa et al., 2016). Developments in sequencing technology have produced long-read sequencing technology or TGS, which offer many advantages over short-read sequencing and can effectively solve the above limitation. As a representative of long-read sequencing, PacBio SMRT sequencing can capture full-length transcripts without the need for further assembly, which makes it an effective method to analyze full-length sequence, AS events, APA sites, lncRNAs, and gene structure at the transcriptome level (Rhoads and Au, 2015; Feng et al., 2019). Furthermore, with continuing progress in accuracy, throughput, and cost reduction, long-read sequencing has become an option for a broad range of applications in genomics and transcriptomics for model and non-model organisms (Amarasinghe et al., 2020).

In this study, we used PacBio SMRT sequencing to assess the Simmental cattle transcriptome by pooling RNA samples from different tissues together. Then, a total of 22.5 Gb of subreads data were obtained and 381,423 CCSs were generated. By detecting the sequences, 276,295 were identified as full-length non-chimeric (FLNC) reads, which accounts for 72.44% of all CCSs. After removing redundant sequences, the consensus sequences were obtained. Meanwhile, we acquired paired-end reads on the Illumina platform, from which ∼981.4 million clean reads were retained after quality filtering. These short reads were subsequently used for correcting the consensus isoform sequences of SMRT sequencing data. Finally, combined SMRT with Illumina data, 22,353 corrected consensus reads were obtained in total. After mapping the consensus reads against the cattle reference genome, the mapping rate was 97.81%, which shows the high quality of the sequencing data.

We used the TAPIS pipeline to perform transcripts structure analysis. After further correction and clustering to eliminate redundancy, we finally got 4,862 high-quality isoforms, among which 2,104 (43.27%) isoforms were classified as known isoforms from known genes, 2,250 (46.28%) were classified as novel isoforms from known genes, and 508 (10.45%) were novel isoforms from novel genes. AS is a crucial transcriptional regulation mechanism for increasing the structural and functional polymorphism of transcripts and proteins. Here, we found 305 AS events using PacBio sequences. The types of most AS were ES and IR. Previous studies have indicated that APA of RNA influenced gene function by changing transcriptome complexity and gene expression. Our study also provides a comprehensive genome-wide APA map draft consisting of 3,795 poly(A) sites from 2,643 genes. These results may underestimate the true number of APA genes because of the low expression of proximal poly(A) sites.

Long non-coding RNAs (LncRNAs) are important regulators of gene expression and are involved in a wide range of biological processes, such as cell proliferation differentiation and modification of chromatin. Several studies have been conducted to identify lncRNAs in cattle, but most of them were performed based on NGS data (Billerey et al., 2014; Koufariotis et al., 2015; Kern et al., 2018). In our study, 569 novel lncRNAs (mean length 1.51 kb) were identified based on PacBio sequencing data. These newly identified lncRNAs will provide additional valid candidates for future functional characterization. Besides, we used the animalTFDB 2.0 database to perform TFs prediction and classification, and then 120 putative TFs from 24 families were identified.

Taken together, our study generated a large number of gene models and alternative isoforms that have not been annotated yet and provide a general encyclopedia of gene transcriptions. These findings refined the annotation of the reference genome and are beneficial for characterizing full-length transcripts of cattle, which are useful for further genetically molecular breeding of cattle. Of course, this profiling of cattle transcriptome would not be exhaustive due to the limited number of sequencing samples.

## Conclusion

Overall, we analyzed the full-length transcriptome of cattle with PacBio SMRT sequencing. Based on full-length transcripts, many AS events, APA sites, novel isoforms, novel lncRNAs, and TFs provide a more comprehensive foundation to explore cattle transcriptome diversity. Our results may provide valuable information for improving cattle draft genome annotation, optimizing the genome structure, and fully characterizing the cattle transcriptome.

## Data Availability Statement

The datasets presented in this study can be found in online repositories. The names of the repository/repositories and accession number(s) can be found below: https://bigd.big.ac.cn/, PRJCA004285.

## Ethics Statement

All animals used in the study were treated following the guidelines established by the Council of China Animal Welfare and were handled in strict accordance with good clinical practices to minimize suffering. Protocols of the experiments were approved by the Science Research Department of the Institute of Animal Sciences, Chinese Academy of Agricultural Sciences (approval number: IAS2020-33). Written informed consent was obtained from the owners for the participation of their animals in this study.

## Author Contributions

JL, HG, and TC designed the study and the interpretation of the findings. LZ, BA, ML, XD, and LD collected the experimental materials and prepared them for the sequencing. TC and BA performed the transcriptome and other computational analyses. WC, BZ, YC, and XG oversaw the project. TC wrote the manuscript. LX revised critically for content and grammar. All authors read and approved the final manuscript.

## Conflict of Interest

The authors declare that the research was conducted in the absence of any commercial or financial relationships that could be construed as a potential conflict of interest.

## Publisher’s Note

All claims expressed in this article are solely those of the authors and do not necessarily represent those of their affiliated organizations, or those of the publisher, the editors and the reviewers. Any product that may be evaluated in this article, or claim that may be made by its manufacturer, is not guaranteed or endorsed by the publisher.

## Supplementary Material

Supplementary Table 1 Prediction of novel isoforms from known genes.

Supplementary Table 2 Prediction of the novel genes.

Supplementary Table 3 Annotation of new genes based on seven databases.

Supplementary Table 4 Results of AS identification.

Supplementary Table 5 Results of poly(A) prediction.

Supplementary Table 6 Prediction of the transcription factors.

Supplementary Table 7 Prediction of the novel lncRNAs.
